# Trichinosis Cured

**Published:** 1881-02

**Authors:** 


TRICHINOSIS CURED.—In a recent issue of the *College and Clinical Record*, Dr. J. M. Barton reports the successful treatment of four cases of Trichinosis with teaspoonfuls or drachm doses of *glycerine*. In view of the now rather frequent occurrence of such cases, the successful treatment detailed by Dr. Barton is worthy of note by practitioners.

